# Unsupervised Drones Swarm Characterization Using RF Signals Analysis and Machine Learning Methods

**DOI:** 10.3390/s23031589

**Published:** 2023-02-01

**Authors:** Nerya Ashush, Shlomo Greenberg, Erez Manor, Yehuda Ben-Shimol

**Affiliations:** 1 School of Electrical and Computer Engineering, Ben Gurion University, Beer-Sheva 84105, Israel; 2 Department of Computer Science, Sami Shamoon College of Engineering, Beer-Sheva 84100, Israel

**Keywords:** drones swarm, radio frequency, wavelet transform, unsupervised clustering, machine learning, dimension reduction

## Abstract

Autonomous unmanned aerial vehicles (UAVs) have attracted increasing academic and industrial attention during the last decade. Using drones have broad benefits in diverse areas, such as civil and military applications, aerial photography and videography, mapping and surveying, agriculture, and disaster management. However, the recent development and innovation in the field of drone (UAV) technology have led to malicious usage of the technology, including the penetration of secure areas (such as airports) and serving terrorist attacks. Autonomous weapon systems might use drone swarms to perform more complex military tasks. Utilizing a large number of drones, simultaneously increases the risk and the reliability of the mission in terms of redundancy, survivability, scalability, and the quality of autonomous performance in a complex environment. This research suggests a new approach for drone swarm characterization and detection using RF signals analysis and various machine learning methods. While most of the existing drone detection and classification methods are typically related to a single drone classification, using supervised approaches, this research work proposes an unsupervised approach for drone swarm characterization. The proposed method utilizes the different radio frequency (RF) signatures of the drone’s transmitters. Various kinds of frequency transform, such as the continuous, discrete, and wavelet scattering transform, have been applied to extract RF features from the radio frequency fingerprint, which have then been used as input for the unsupervised classifier. To reduce the input data dimension, we suggest using unsupervised approaches such as Principal component analysis (PCA), independent component analysis (ICA), uniform manifold approximation and projection (UMAP), and the t-distributed symmetric neighbor embedding (t-SNE) algorithms. The proposed clustering approach is based on common unsupervised methods, including K-means, mean shift, and X-means algorithms. The proposed approach has been evaluated using self-built and common drone swarm datasets. The results demonstrate a classification accuracy of about 95% under additive Gaussian white noise with different levels of SNR.

## 1. Introduction

The rapid proliferation of technology has increased the use and capabilities of autonomous systems. The ability to perform remote tasks has been found to be more available. To this end, drones are a suitable platform for performing unmanned missions. While the reliability of a single drone in performing long and autonomous tasks is limited, a swarm of drones is more reliable for performing more complex missions. In accordance, the use of multiple drones is growing, and applications that primarily benefit from this new technology include the cooperation method for target searching in unknown environments [1,2], data collection platform [3,4] and sensing applications for civilian uses such as gas-seeking, smart agriculture, and goods delivery. In particular, the exploitation of UAVs for many applications has received considerable attention in many fields [5,6,7]. An autonomous multi-UAV system also benefits traditional UAV-based solutions, such as the speed of performing tasks, and increasing the speed of data collection from inaccessible places. These advantages can be exploited to use UAVs swarm for tactical purposes, such as attacking multiple targets [8] or creating distractions in airports [9].

This paper proposes an unsupervised method for drone swarm characterization using RF analysis and providing the early detection of a drone swarm attack by estimating the number of drones in the swarm. We assume that the drone types and the number of drones in the swarm are unknown. The proposed detection approach is based on the RF signature derived from internal communications between the drones.

The proposed method includes the following main phases: (a) creating a dataset based on swarm RF communication, (b) using preprocessing techniques to normalize the data and remove anomalies phenomenon, (c) extracting wavelet-based features from the RF signals, and (d) using dimension reduction and clustering algorithms for classification.

The main contributions of this paper are:

- Developing a novel method for unsupervised drone swarm characterization and detection using RF signals and machine-learning algorithms with no a priori knowledge and no labeled data.
- We propose an efficient way to assess the number of drones in a swarm and the risk that comes from automated UAVs beforehand.
- An evaluation of the proposed approach on common datasets published in the literature.
- A comparison of the performance using various features, such as WST and CWT, and different dimension reduction methods.

The rest of this paper is organized as follows: Section 2 reviews related work, Section 3 presents the proposed approach, Section 4 shows the experimental results, and Section 5 concludes the paper.

## 2. Background and Related Work

This section presents recent relevant studies that are related to single drone detection. The recent studies are based on visual images, radar, audio, and RF.

S. Singha and B. Aydin [10] proposed an image-based method using a convolutional neural network (CNN) for drone classification. The authors used YOLOv4 CNN architecture to detect multiple objects and achieve an accuracy of 95%. They used a common dataset which included 2395 images of drones and birds. Similar works which use image-based drone classification and CNN demonstrate an average accuracy of 80–90% [11,12,13,14].

R. Fu et al. [15] presented drone classification at millimeter-wave (mmWave) radars using deep learning techniques. The authors used a long short-term memory (LSTM) network and an adaptive learning rate optimizing (ALRO) model to train the LSTM. The proposed LSTM-ALRO model can work well under a highly uncertain and dynamic environment. They achieved an accuracy of 99.88%. Similar works based on the radar for drone classification in different radar systems (1 GHz–24 GHz) can achieve a high accuracy of 95–100% using machine learning methods [16,17,18,19,20].

S. Al-Emadi [21] proposed drone detection and identification processes using the drone’s acoustic features with different deep learning algorithms, namely, the CNN, the recurrent neural network (RNN), and the convolutional recurrent neural network (CRNN) in drone detection and identification. They used a common dataset [22] and a generative adversarial network (GAN) technique for artificial data generation to generate a large artificial drone acoustic dataset to improve drone presence detection. The experiment has shown that the results of both CNN and CRNN are outstanding with accuracy, precision, recall, and F1 score with values higher than 90%. Other works [23,24] achieved an accuracy of 83% and 98.97%, respectively, using audio signals and machine learning methods. A swarm of drones contains several drones that are characterized by mixed-emitted audio. Z. Uddin et al. [25] suggested a method for detecting multiple drones in a time-varying scenario using acoustic signals by ICA, SVM, and KNN.

O. Medaiyese et al. [26] performed a thorough analysis of an RF-based drone detection and identification system (DDI) under wireless interference, such as WiFi and Bluetooth. The radio pulses of communication between the UAV and its flight controller could be intercepted as an RF signature for UAV detection. Using these RF signals as signatures is based on the premise that each UAV-flight controller communication has unique features that are not necessarily based on the modulation types or propagating frequencies but may be a result of imperfection in the communication circuitries in the devices. O. Medaiyese et al. [26] achieved an accuracy of 98.9% at 10 dB SNR while using machine learning algorithms and a pre-trained convolutional neural network-based algorithm called SqueezeNet, as classifiers. More RF-based works [27,28,29,30] presented high accuracy (above 95%) results using the RF signals emitted from the drones while extracting features such as wavelet and PSD to train the machine learning algorithms. Since a swarm of drones is characterized by using the same drone type, the problem is more complex. N. Soltani et al. [31] provided a UAV classification of the same model. They used seven identical DJI Matrice 100 UAVs at different distances from a receiver, which was recorded, and the authors built a multi-classifier-based neural network to reveal unseen drones with an accuracy of 99% on M100 dataset. We utilized the same dataset in our work.

Internal wireless communications for drones are essential since they allow drones to operate without being tethered to a ground-based control system. The escalating use of drones presents also safety problems demanding data protection and cyber security. Wang et al. [32] analyzed cybersecurity efficiency to incorporate compelling security features into wireless communication systems. Since the communication of drones has become more secure, there is a significant advantage to the characterization and identification of a swarm of drones using only RF signals without reference to the content of the communication.

## 3. Proposed Approach

This section presents the problem statement and the proposed approach. We suggest using unsupervised drone swarm characterization and detection based on RF signal analysis and machine learning methods. The proposed method utilizes different radio frequency fingerprints (RFF). V. Brik et al. [33], showed how each transmitter had a unique RFF which arose from imperfections in the analog components during the manufacturing process.

**Problem statement:** Let us consider that N drones are denoted as $D_{1},D_{2},\ldots ,D_{N}$ and communicate using RF packets while assuming that the communication protocol is unknown. Each RF transmitter sends multiple packets *p* with an unknown dimension $p\in R^{d_{i}}$ (i.e, with various length). This research proposed an unsupervised method that could match each transmitted packet to the specific drone that sent it, assuming no apriori knowledge about the number of drones, while each drone might send a different number of messages. Let us consider *m* packets from different drones $p_{1},p_{2},\ldots ,p_{m}$ when *m* depicts the total number of sent messages. We aim to estimate the number of drones (in the swarm), which is equivalent to the number of RF transmitters.

Assuming that $l_{i}$ stands for the number of packets transmitted from drone *i* and $l_{i}\geq 1$, Equation (1) depicts that packets ${\left\{p_{j}\right\}}_{j=1}^{l_{i}}$ belong to drone $D_{i}$ where 0<i≤N. Where *i* is one drone from the swarm, $D_{1},D_{2},\ldots ,D_{N}$.

(1) $${\left\{p_{j}\right\}}_{j=1}^{l_{i}}\in D_{i}$$

**Figure 1:**
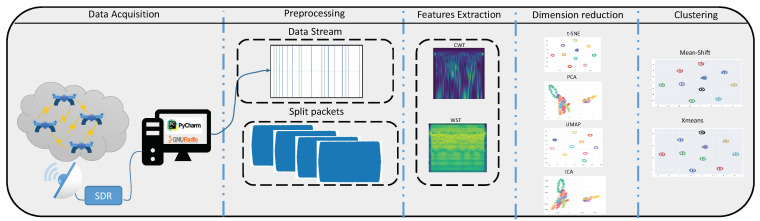
Proposed method pipeline.

Most of the published studies relate to single drone detection using both supervised and unsupervised approaches, while we propose an unsupervised approach that aims to detect multiple drones. To the best of our knowledge, this is the first work that applies unsupervised learning for discriminating RF packets (belonging to different drones), identically classifying multiple drones using an unsupervised approach, and estimating the number of drones in the swarm. The advantage of this approach is that no labeled data or pre-training is needed for drone swarm classification. In addition, no apriori knowledge of the drone type is needed; therefore, the RF transmitter might be the same for all the drones, which makes the classification problem harder. Figure 1 depicts the main stages of the proposed approach, including the creation of the dataset, preprocessing, features extraction, dimension reduction, and clustering, as described in the following sections.

### 3.1. Datasets

This section describes the RF datasets we used in order to evaluate the approach during this research. G. Vásárhelyi et al. and F. Hu et al. suggest using the ZigBee protocol for drone-to-drone communication in a physical scenario of swarm drones [34,35]. The ZigBee protocol supports a large number of nodes and is energy efficient. Therefore our self-build dataset is based on ZigBee as described in Section 3.1.1. In addition to the self-built XBee dataset, we used several datasets published in the literature described in Section 3.1.2.

#### 3.1.1. Self-Built Dataset

The dataset includes 10 XBee ZB S2C based on ZigBee communication, where one serves as a coordinator device. All the XBee modules are configured with the same properties. We used a GNU radio platform [36] with SDR for acquire the RF signals.

#### 3.1.2. Common Dataset

The common RF dataset we used in this research is as follows. (1) We adapted the RF datasets provided by N. Soltani et al. [31]. This dataset contains the data acquired from seven identical drones (DJI Matrice 100 UAVs). The dataset contains 13k examples representing RF signals from the drones. (2) Allahham et al. [37] suggested another drone RF dataset which included three drones: Phantom, AR, and Bepop. They present an RF-based dataset of drones functioning in different modes. The dataset consists of recorded segments of RF background activities with no drones and segments of drones operating in different modes such as off, on, connected, hovering, flying, and video recording. (3) M. Ezuma [38] presented datasets containing RF signals from drones and remote controllers (RCs); the drones recorded in this dataset are Inspire, Matrice, and Phantom. (4) The dataset provided by E. Uzundurukan et al. [39] consisted of Bluetooth (BT) signals collected at different sampling rates from 27 different smartphones (six manufacturers with several models for each). We suggest using the M100 dataset described in one separately from the mixed datasets derived from the 2–4 (VRF dataset).

### 3.2. Feature Extraction

Unique radio frequency characterizes each RF transmitter (representing a drone). Fingerprints (RFF) are due to the nonlinear component of each transmitting device. The feature extraction is based on two types of wavelet transform. The wavelet transform is widely used as an efficient feature due to its time-frequency localization properties. We used two kinds of wavelet transform: CWT [40] and WST [41].

**Figure 2:**
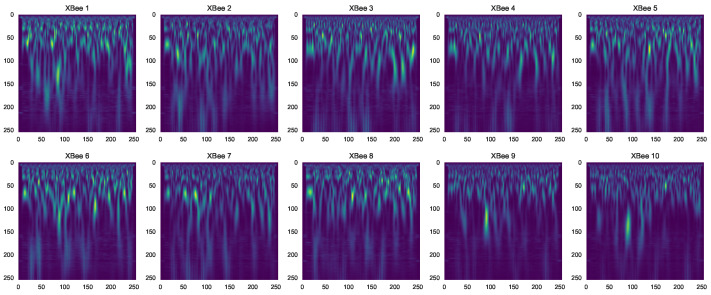
CWT of each XBee transmitter.

**Continuous Wavelet Transform (CWT)**—The CWT originally introduced by P. Goupillaud et al. [42] was used to analyze signals at different scales or resolutions. The wavelet function was scaled and translated in order to analyze the signal at different scales and locations. This allowed the CWT to provide information about the frequency components of the signal at different scales, which could be useful for identifying patterns or features in the signal that might not be apparent at the time domain. The absolute value of the CWT, the so-called scalogram, is expressed by Equation (2). Figure 2 shows the scalogram images of the CWT transform for various RF transmitters.

(2) $$SC_{x}\left(a,\tau \right)=|\Psi_{a,\tau (t)}{|}^{2}=\frac{1}{\left|a\right|}{\left|\int_{-\infty }^{\infty }x\left(t\right)\hat{\Psi }\left(\frac{t-\tau }{a}\right)\right|}^{2}$$

where, x(t) is the 1D signal to be transformed. τ is the translation parameter, *a* is the scale (or dilation) parameter of the wavelet function, and Ψ is called the mother wavelet.

**Figure 3:**
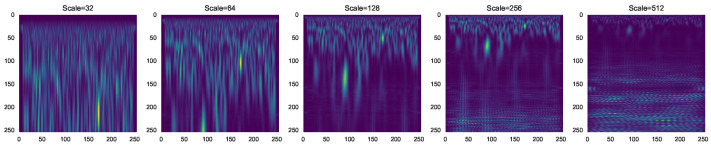
CWT scalogram in different scales.

Figure 3 shows the CWT images for various scales. For large-scale, we obtained low-frequency information and for small-scale high-frequency, the information is presented in the CWT images.

**Figure 4:**
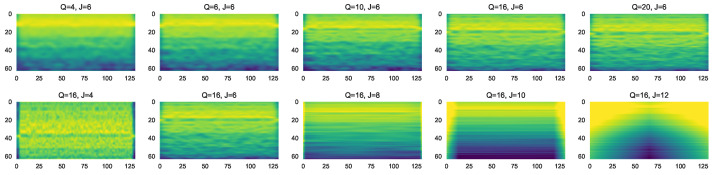
WST decomposition of Xbee signal.

**Wavelet Scattering Transform (WST)**—Wavelet scattering transform [43] is used to extract discriminant features from the RF-signals. WST refers to an iterative process of applying a set of wavelet transforms and nonlinearities at different scales, making it stable in the case of small deformations and invariant to the input signal translations or rotations. The WST transformation was carried out using (1) convolution, (2) nonlinearity, and (3) averaging. Precisely, the WST coefficients are obtained by applying the convolution operator ∗ between the wavelet modulus and low-pass filter ϕ. Assuming that wavelet ψ(t) is a bandpass filter with a central frequency normalized to one at time index *t*, the wavelet filter bank $\psi_{\lambda }\left(t\right)$ is defined in Equation (3) as follows:

(3) $$\Psi_{\lambda }\left(t\right)=\lambda \Psi \left(\lambda t\right)$$

where $\lambda =2^{\frac{J}{Q}}$ and *Q* define the number of wavelets that are used in one octave of the frequencies. The bandwidth of the wavelet Ψ(t) is of the order $\frac{1}{Q}$, and as a result, the filter bank is composed of bandpass filters that are centered in the frequency domain in λ and have a frequency bandwidth of $\frac{\lambda }{Q}$. Figure 4 depicts the WST decomposition for different values of *Q* and J.

**Figure 5:**
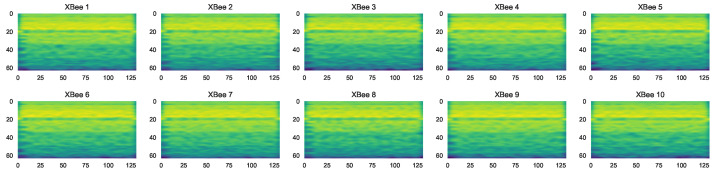
WST from different 10 XBee modules.

Figure 5 shows the WST transform for various RF transmitters. We set *Q* = 16 and J = 6; other different settings have been tried for the invariance scale and wavelet octave resolution, but this architecture preserves the signal information that is best for our case.

### 3.3. Dimension Reduction

The dimension reduction process helps us to pull together only clusters corresponding to the same transmitter. The correlation between the samples indicates that they came from the same source; we applied some different types of dimension reduction methods, linear (PCA, ICA) and nonlinear (t-SNE, UMAP), and compared the results using each. The nonlinear methods are graph-based, creating a high-dimensional graph and then reconstructing it in a lower-dimensional space while retaining the structure.

**t-Distributed Stochastic Neighbor (t-SNE)**—t-SNE is a nonlinear approach for dimension reduction [44] and is used to model pairwise similarities between points in both higher dimensional $p_{\left(i|j\right)}$ and lower dimensional spaces $q_{\left(i|j\right)}$. Therefore, if two points $x_{i}$ and $x_{j}$ are close in the input space, then their corresponding points $y_{i}$ and $y_{j}$ are also close. Equation (4) describes the affinities between points $x_{i}$ and $x_{j}$ in the input space $p_{ij}$.

(4) $$p_{\left(i|j\right)}=\frac{\exp(-\frac{\left|\right|x_{i}-x_{j}{\left|\right|}^{2}}{2\sigma_{i}^{2}})}{\sum_{k\neq i}\exp\left(-\frac{\left|\right|x_{i}-x_{k}{\left|\right|}^{2}}{2\sigma_{i}^{2}}\right)}$$

where $\sigma_{i}$ is the bandwidth of the Gaussian distribution, and it is chosen using the perplexity of $P_{i}$. Perplexity can be defined as the smooth measure of an effective number of neighbors $prep\left(P_{i}\right)=2^{H(p_{i})}$. $P_{i}$ is the conditional distribution of all the other points given $x_{i}$. Similarly, Equation (5) shows that the affinity between points $y_{i}$ and $y_{j}$ in the embedding space can be defined using the Cauchy kernel.

(5) $$q_{\left(i|j\right)}=\frac{\exp(-||y_{i}-y_{j}|{|}^{2})}{\sum_{k\neq i}\exp(-||y_{i}-y_{k}{\left|\right|}^{2})}$$

t-SNE finds the points $\left\{y_{1},\ldots ,y_{n}\right\}$ that minimize the Kullback–Leibler (KL) divergence between the joint distribution of points in the input space P and the joint distribution of the points in the embedding space Q. To minimize the KL-divergence, starting with the random initialization, the cost function C(Y) described in Equation (6) is minimized by gradient descent.

(6) $$\frac{\delta C}{\delta y_{i}}=4\sum_{j}\left(p_{ij}-q_{ij}\right)\left(y_{i}-y_{j}\right)\left(1+|\right|y_{i}-y_{j}{\left|\right|}^{2}{)}^{-1}$$

**Uniform Manifold Approximation (UMAP)**—UMAP uses local manifold approximations and assembles together their local fuzzy-simplicial set representations to form a topological representation of the high-dimensional data. Given some low-dimensional representations of the data, the layout of the data representation in the low-dimensional space is then optimized through the minimization of the cross-entropy between the two topological representations [45]. The cost function for the optimization process, which is carried out by minimizing the fuzzy-set cross-entropy, is depicted by Equation (7) as follows:

(7) $$C_{UMAP}=\sum_{i\neq j}v_{ij}\log\left(\frac{v_{ij}}{w_{ij}}\right)+\left(1-v_{ij}\right)\log\left(\frac{1-v_{ij}}{1-w_{ij}}\right)$$

where $v_{ij}$ refers to the local fuzzy simplicial set memberships defined in the high-dimensional space on the basis of the smooth nearest-neighbors distances, whereas $w_{ij}$ refers to the low-dimensional similarities between *i* and *j*. For the UMAP optimization, stochastic gradient descent is used to minimize the cost function.

**Principal Component Analysis (PCA)**—PCA is used to transform data linearly into a low-dimensional subspace by obtaining the maximized variance of the data. The resulting vectors are an uncorrelated orthogonal basis set, where the principal components are the eigenvectors of the symmetric covariance matrix of the observed data. Using PCA for dimension reduction should retain the extracted principal components corresponding to the *m* eigenvalues from the total eigenvalues, where $\gamma_{k}$ is called the percentage retained in the data representation as described in Equation (8).

(8) $$\gamma_{k}=\frac{\lambda_{1}+\lambda_{1}+\ldots +\lambda_{m}}{\lambda_{1}+\lambda_{1}+\ldots +\lambda_{m}+\ldots +\lambda_{k}}$$

**Independent Component Analysis (ICA)**—ICA is a statistical and computational technique that is used to extract features from a set of measurements, such as when the features are maximally independent. The observed variables $x_{1}\left(t\right),x_{2}\left(t\right),\ldots ,x_{n}\left(t\right)$ are composed of a linear combination of original and mutually independent sources $s_{1}\left(t\right),s_{2}\left(t\right),\ldots ,s_{n}\left(t\right)$ at time point *t* as defined in Equation (9).

(9) x(t)=As(t)

where *A* is a mixing matrix with a full rank. Equation (10) describes the independent vector components of the ICA.

(10) y=Wx

where $W=A^{-1}$ is the demixing matrix and $y=y_{1},y_{2},\ldots ,y_{n}$ denotes the independent components. The task is to estimate the demixing matrix and independent components only based on the mixed observations, which can be conducted by various ICA algorithms such as fastICA, JADE, Infomax, etc. Section 4 describes the dimension reduction results using all the algorithms mentioned above on the RF signals.

### 3.4. Clustering

We suggest using an unsupervised method to estimate the number of clusters. When using mean-shift and xmeans, we estimated the number of clusters and implemented it using Scikit-learn [46], and PyClustering [47].

**Mean-Shift**—The mean-shift algorithm is an unsupervised clustering algorithm that seeks to find dense areas of data points in a dataset [48]. An important characteristic of the mean shift is that it does not require prior knowledge of the number of clusters and does not constrain the shape of the clusters. The number of clusters is determined by shifting the data points iteratively toward the mean until convergence is achieved. Given *n* data points $x_{j}\left(j=1,\ldots ,n\right)$ in the *d*-dimensional space $R^{d}$, the mean shift vector at point *x* is defined in Equations (11) and (12).

(11) $$M_{g,h}\left(x\right)=\frac{\sum_{j=1}^{n}x_{j}g(||\frac{x-x_{j}}{h}{\left|\right|}^{2})}{\sum_{j=1}^{n}g(||\frac{x-x_{j}}{h}{\left|\right|}^{2})}-x$$

where *h* is the bandwidth parameter and *g*(·) is called the profile of the kernel G(x).

(12) $$x\left(t+1\right)\leftarrow x\left(t\right)+M_{h,g}\left[x\left(t\right)\right]$$

where *t* denotes the iteration number, the iterative process converges toward the local maxima. The mean shift vector always points toward the direction of the maximum increase in density, and successive vectors can define a path leading to a mode of the estimated density. All data points that have converged to the same mode are grouped together as a cluster. In the mean shift theory, a cluster is defined as an area of higher density than the remainder of the dataset, and a dense region in the feature space corresponds to a mode (or a local maximum) of the probability density distribution. The ultimate result of the mean shift procedure associates each point with a particular cluster [49].

**X-Means**—The X-means algorithm is a k-means extension that can be used to estimate the number of clusters [50]. Cluster centers are split locally during each iteration of the k-means algorithm to obtain better clustering. Splitting decisions are based on the Bayesian Information Criterion (BIC) or Akaike Information Criterion (AIC) as described in Equations (13) and (14).

**Figure 6:**
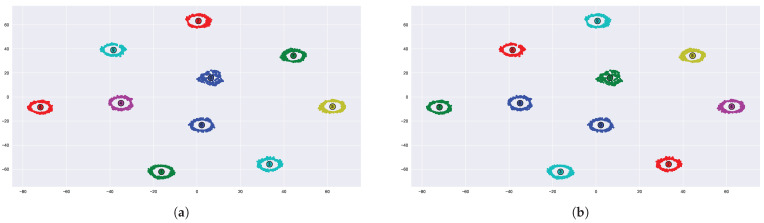
Estimate the number of clusters using mean-shift (**a**) and x-means (**b**).

(13) BIC=−2∗LL(N)+log(N)∗k

(14) AIC=−2∗LL(N)+2k

where *N* is the number of samples, LL(N) is the log-likelihood as a function of *N*, and *k* is the number of parameters in the model. Figure 6 shows the estimation of K clusters using the x-means and mean-shift for the XBee’s signals after dimension reduction with the t-SNE algorithm. We have full cluster separation here, and both mean-shift and x-means work well and estimate 10 clusters based on the given points.

**Figure 7:**
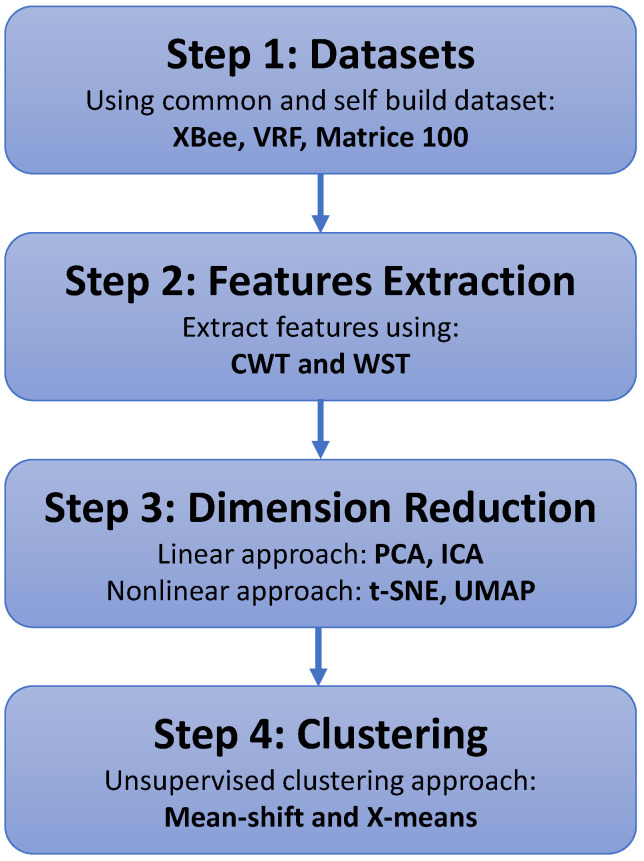
Flowchart of the proposed method.

To summarize the whole process, the main stages are presented in a flowchart as described in Figure 7.

## 4. Experimental Results

This section describes the experimental results for the proposed approach using CWT and WST wavelet features. The proposed method has been evaluated with the five datasets described in Section 3.1. The results show the efficiency of the approach and demonstrate good discrimination between the different RF sources. The number of drones in the swarm was accurately detected for all the tested datasets, demonstrating a success rate of around 95%. We applied various dimension reduction methods (t-SNE, UMAP, PCA, and ICA) and two unsupervised clustering methods: mean-shift and x-means. This chapter is organized as follows: Section 4.1 evaluates the proposed method for various RF sources (VRF dataset), which include four different types of drones and two other RF sources (smartphones) taken from [37,38,39]. Section 4.2 shows the results for the XBee dataset, which contains 10 identical transceivers based on the ZigBee communication protocol, which was self-acquired in our lab. Finally, Section 4.3 shows the results for the Matrice dataset taken from [31], which contains an RF dataset derived from seven identical Matrice 100 (M100 dataset) drones.

### 4.1. Various RF Sources (VRF Dataset)

**Figure 8:**
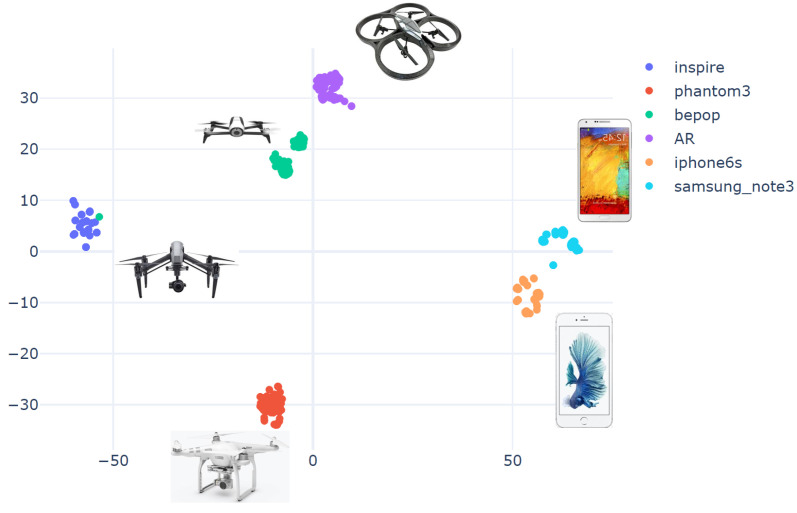
Cluster results for WST using t-SNE.

This section demonstrates the efficiency of the proposed method for different types of RF transmitters. We used the VRF dataset as a basic experiment to prove the concept of the proposed unsupervised clustering method, which was applied to various RF sources. The dataset included RF data acquired from four different drones [37,38,39]: Inspire, Phantom, Bepop, AR, and two more RF sources (cellular phones): “IPhone 6S” and “Samsung Note”. Figure 8 depicts the clustering results for the six different RF sources (using WST and t-SNE) for 100 samples from each source. The figure represents the 2D projection of the wavelet transform (WST), where each point represents a single RF packet. RF packets transmitted from the same drone have the same color in the graph (for example, green stands for Bepop drone). It can be seen that an almost perfect clustering was achieved while using WST and t-SNE.

#### 4.1.1. Clustering Accuracy Criteria (CAC)

We suggest using the clustering accuracy criteria based on the common k-means unsupervised clustering algorithm [51]. The K-means algorithm is applied to the 2D wavelet domain after using t-SNE for dimension reduction. It is assumed that the number of sources (i.e., the number of clusters) is apriori known (k = 6 for this dataset). The CAC criteria, i.e., the number of correctly classified samples divided by the total samples, is defined by Equation (15).

(15) $$Clusters\;accuracy=\frac{True\;samples\;position}{Total\;samples}$$

The CAC accuracy for the VRF dataset is 99% since only one sample was wrongly classified (the ’Bepop’ sample (green) was wrongly classified as an Inspire (blue) drone), as shown in Figure 8.

#### 4.1.2. Estimating the of Number of Clusters

**Figure 9:**
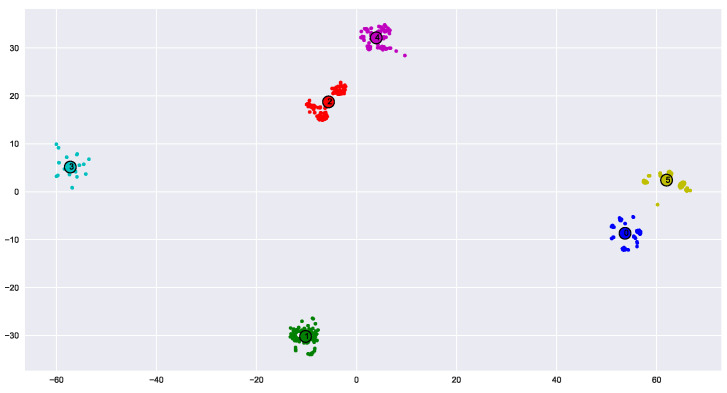
Mean shift for unsupervised clustering.

To automatically extract the number of clusters, we suggest using one of the unsupervised clustering methods, mean-shift or x-means. Figure 9 shows the results of the mean-shift unsupervised clustering for the VRF dataset (similar results were achieved while using X-means). The results show that exactly six clusters were found. The center of each cluster is marked by a small circle.

### 4.2. XBee Dataset

**Figure 10:**
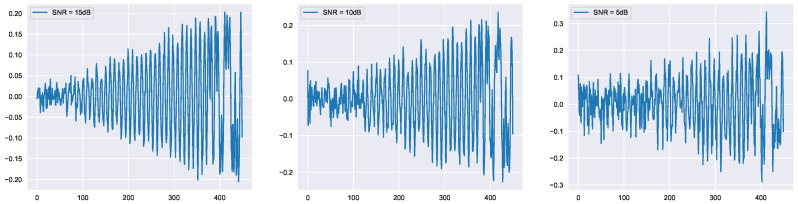
XBee’s RF signal with different SNR values.

We evaluated the proposed method for the XBee self-dataset acquired in our lab. The XBee dataset contains ten identical XBee transmitters. Adding additive white gaussian noise (AWGN) was evaluated for various SNRs to examine the robustness of the proposed approach. The dataset included about 240 samples per XBee transmitter. Figure 10 depicts the effect of AWGN on the RF signals for different SNR values (for the RF transient state).

**Figure 11:**
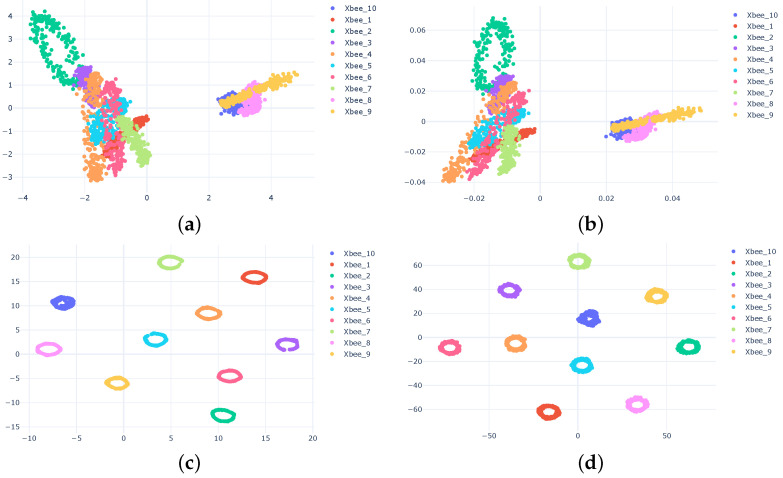
Clustering results using PCA (**a**), ICA (**b**), UMAP (**c**) and t-SNE (**d**).

The rest of this section shows the clustering results for the noisy XBee dataset. Figure 11 shows the clustering results for the four dimension reduction methods (t-SNE, UMAP, ICA, and PCA) using CWT features without adding noise. The results show that using linear approaches, such as ICA and PCA, does not provide good separation. However, the t-SNE and UMAP (which are both nonlinear) provide very good separations. Therefore, we adapted the t-SNE and the UMAP as favorite approaches for dimension reduction.

**Figure 12:**
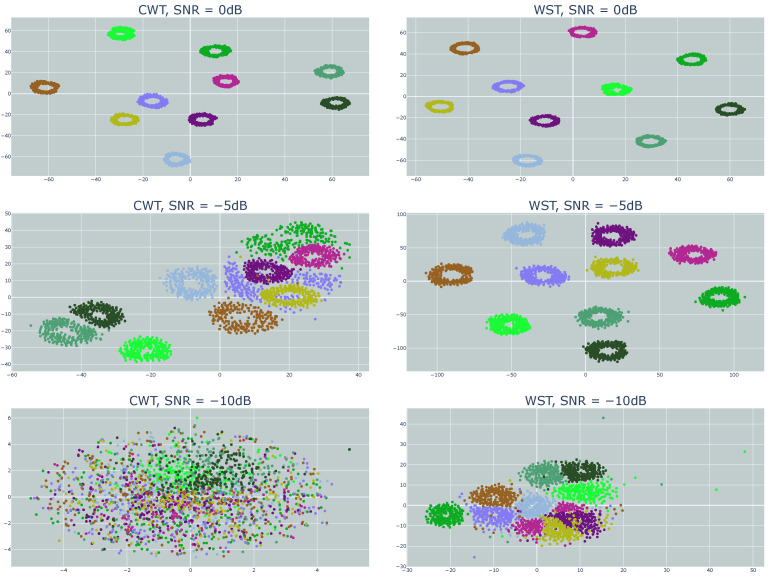
Clustering results using t-SNE with WST and CWT for different SNR values.

**Figure 13:**
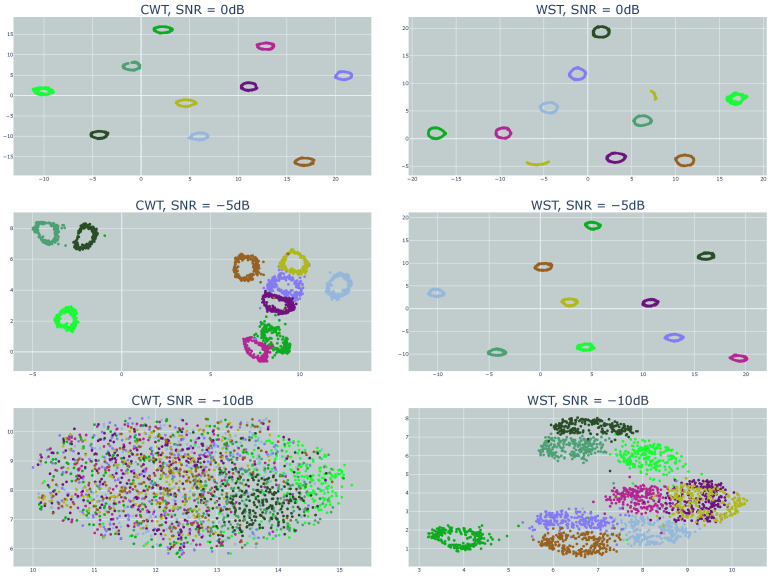
Clustering results using UMAP with WST and CWT for different SNR values.

Figure 12 and Figure 13 show the clustering results for different SNR values using t-SNE and UMAP, respectively. For both techniques, the WST outperformed the CWT. While a good cluster separation was achieved using WST for the SNR of −5 dB, the CWT failed to separate the clusters, which was even more emphasized for the SNR of −10 dB.

Figure 13 shows the results using UMAP as a dimension reduction. While a good cluster separation was achieved using WST for the SNR of −5 dB, the CWT failed to fully separate the clusters, and part of them became mixed together. This was even more emphasized for the SNR of −10 dB. A good separation was achievable under a wide range of AWGN with both t-SNE and UMAP. WST was more immune to noise and provided a good cluster separation for low SNR.

**Figure 14:**
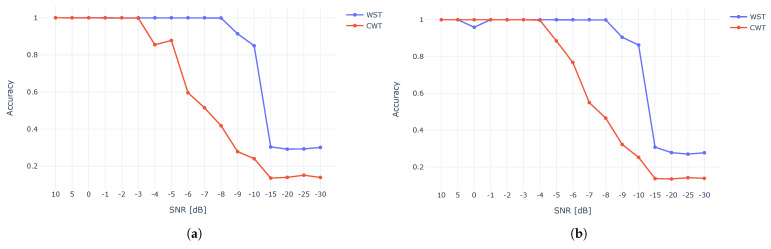
Clustering accuracy using t-SNE (**a**) and UMAP (**b**) for CWT and WST for different SNR.

To evaluate the success rate of the XBee dataset under AWGN, we used CAC criteria. We assumed that the number of clusters was apriori known (ten in this case). Figure 14 depicts the clustering accuracy for different SNR values. The results show that WST outperformed the CWT and was more immune to noise, demonstrating 80–100% up to about −10 dB for both t-SNE and UMAP, while CWT demonstrated an over 80% accuracy up to about −5 dB.

**Figure 15:**
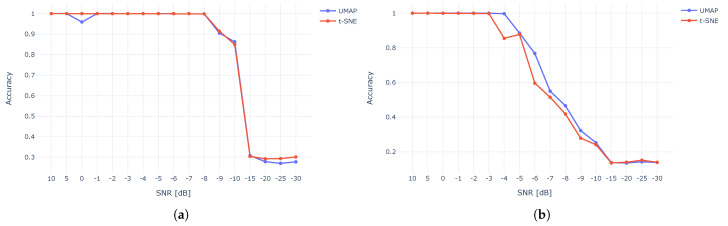
t-SNE and UMAP accuracy for WST (**a**) and CWT (**b**).

Figure 15 compares the accuracy of t-SNE against UMAP. The results show a very similar accuracy for both methods while using WST (Figure 15a) and CWT (Figure 15b).

**Figure 16:**
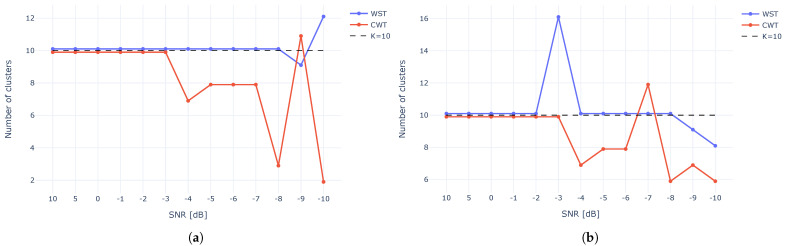
Number of clusters using mean-shift (**a**) and x-means (**b**) for various SNR.

Figure 16 shows the results of mean-shift (Figure 16a) and x-means (Figure 16b) clustering techniques for both WST and CWT as a function of SNR. Although both clustering methods show similar results, and the mean shift is more accurate, the estimation of the number of clusters is perfect (10 clusters) up to −8 dB for using WST and mean-shift while the x-means wrongly estimated the number of clusters at −3 dB. For CWT, we perfectly estimated the clusters up to −3 dB for both the mean-shift and x-means. WST was more accurate and outperformed CWT.

### 4.3. Matrice Dataset

**Figure 17:**
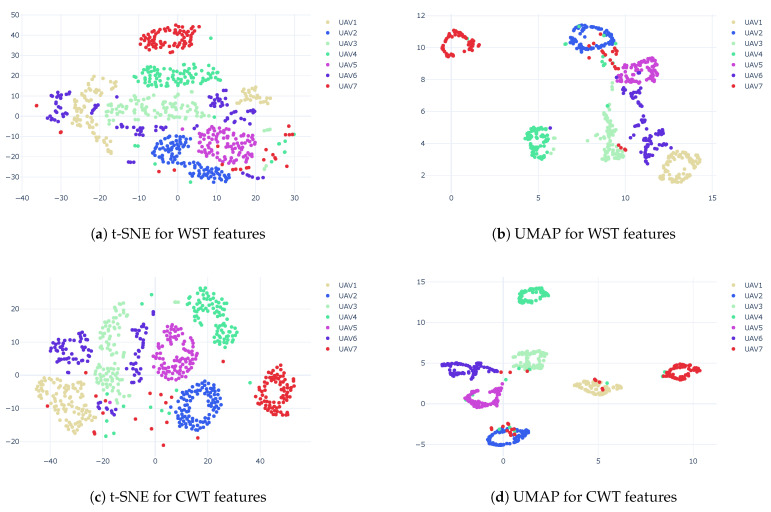
Clustering results using t-SNE and UMAP for both WST (**a**,**b**) and CWT (**c**,**d**).

N. Soltani et al. [31] provided an RF dataset that contained packets from 7seven identical Matrice 100 drones (M100 dataset). Figure 17 shows the clustering results for the M100 dataset using t-SNE and UMAP for both WST and CWT features. UMAP outperformed t-SNE, and CWT outperformed WST. The results using CAC show that for t-SNE, we achieved an accuracy of 60% and 75% for WST and CWT, respectively; for UMAP, we obtained an accuracy of 90% and 95% success rates for WST and CWT, respectively.

**Figure 18:**
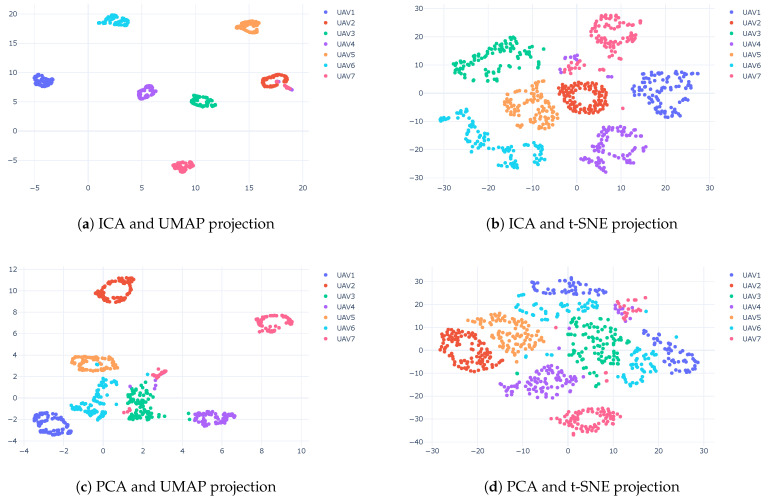
Clustering results using linear and nonlinear techniques for WST features.

To improve the clustering results, we suggest using a linear technique (ICA or PCA) for pre-dimension reductions before applying UMAP or t-SNE. In the pre-dimensional reduction phase, the most valuable principal components were extracted, and the less important information was removed. Figure 18 shows that using both linear and nonlinear methods increased the accuracy. It can be seen that ICA outperformed PCA, and UMAP outperformed t-SNE. The results show that using CAC for t-SNE achieved an accuracy of 75% while using PCA and ICA as pre-dimension reductions produced an accuracy of 95%, respectively. For UMAP, we achieved an accuracy of 92% and 95% for PCA and ICA as the pre-dimension reduction, respectively. The best result was achieved using ICA and UMAP.

**Figure 19:**
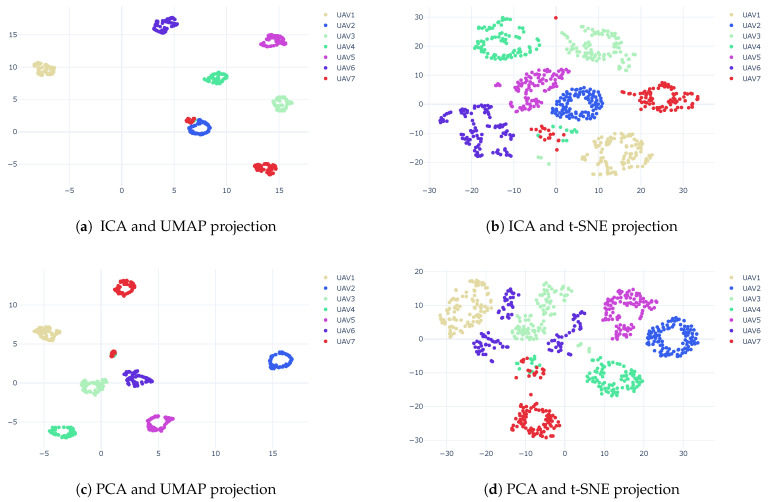
Clustering results using linear and nonlinear techniques for CWT features.

Figure 19 shows that using CWT with pre-dimension reduction provides the best result for clustering. The results show that for t-SNE, we achieved an accuracy of 81% and 94% using PCA and ICA as the pre-dimension reduction, respectively, and for UMAP, we achieved an accuracy of 95% for both PCA and ICA as the pre-dimension reduction.

**Figure 20:**
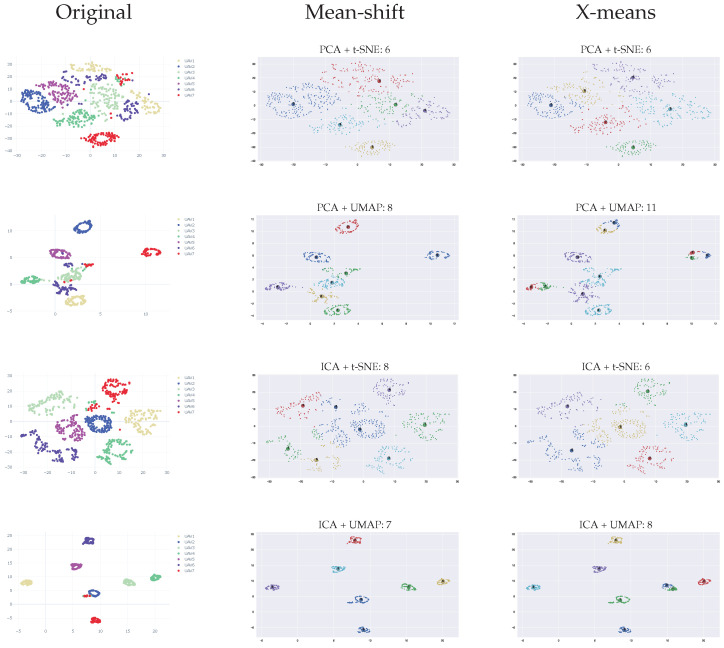
Clustering results using WST and various dimension reduction methods.

**Figure 21:**
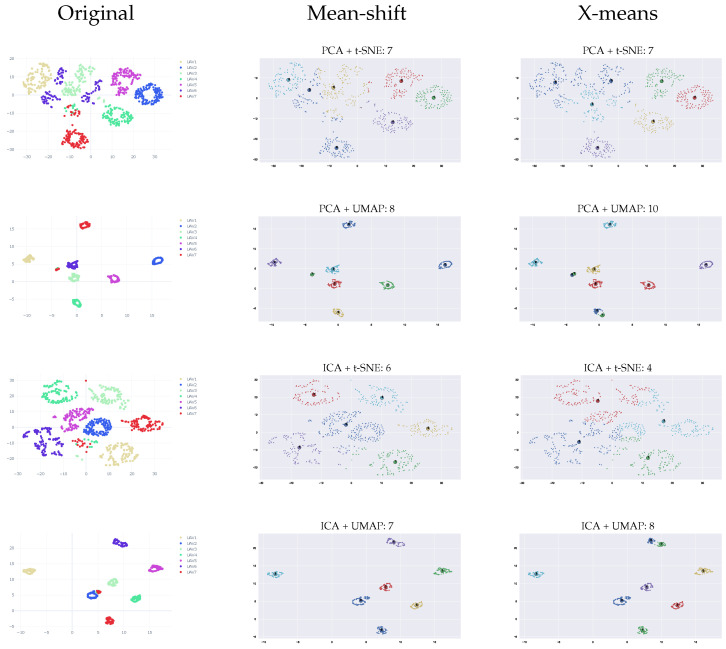
Clustering results using CWT and various dimension reduction methods.

Figure 20 and Figure 21 depict the number of clusters estimation using both mean-shift and x-means for all the dimension reduction techniques. The results show that for the M100 dataset, CWT outperforms WST, with an average accuracy of 91% in comparison to 89%. mean-shift outperforms x-means and can identify clusters with high accuracy when the clusters are well separated. While using x-means does not necessarily produce an accurate result, even with good separation between the clusters.

## 5. Summary and Conclusions

This research presents an unsupervised machine learning-based approach for drone swarm characterization and detection using RF signals analysis. In contrast to the existing studies applied to RF-based drone detection, we suggest using an unsupervised approach. As far as we know, this is the first time that an unsupervised-based approach with no a-priory knowledge regarding the drone’s RF signature has been successfully applied for detecting and clustering different types of unknown drones. This work suggests analyzing time series signals using wavelet transforms, specifically, the CWT and WST transforms, to extract RF signature features. To reduce the multidimensional space of the extracted wavelet features, we propose using both linear (PCA and ICA) and nonlinear (t-SNE and UMAP) common dimension reduction methods. The drone clustering and estimation of the number of drones in the swarm are successfully carried out in the low-dimension space using methods such as Mean-shift and X-means. One of the main contributions of this research is that no labeled data nor pre-training is needed for the detection and classification of drones in a swarm. Therefore, the proposed approach needs no prior knowledge and does not depend on the drone type. The results show that using linear approaches such as ICA and PCA does not provide a clear separation in the low dimension, while the nonlinear approaches, including t-SNE and UMAP, provide a good and accurate classification based on the wavelets features in the low-dimension space. The proposed method has been applied to various datasets, including the RF data sets acquired in our Lab (the VRF and XBee) and the published common Matrice dataset. The results demonstrate a success rate of 99% for the VRF dataset and 100% for the XBee dataset for SNR up to −8 dB while using WST features. For most of the tested scenarios, WST features outperformed the CWT features. An average accuracy of 95% was achieved for the common Matrice dataset. The best cluster separation was achieved using a combination of both ICA as a linear pre-dimension reduction method and the nonlinear UMAP for post-dimension reduction. Future work may focus on integrating and testing deep learning methods for automatic drone signature feature extraction and accurate classification in noisy environments.

## Data Availability

Not applicable.
